# Notch regulates *Histoplasma capsulatum* clearance in mouse lungs during innate and adaptive immune response phases in primary infection

**DOI:** 10.1002/JLB.4A1221-743R

**Published:** 2022-05-23

**Authors:** Shuo Huang, George S. Deepe

**Affiliations:** ^1^ Department of Internal Medicine and Department of Pathology, Pathobiology and Molecular Medicine Program University of Cincinnati College of Medicine, Cincinnati, USA; ^2^ Department of Medicine, Division of Infectious Diseases, University of Cincinnati College of Medicine, Cincinnati, Ohio, USA 234 Albert Sabin Way Cincinnati OH 45267 United States

**Keywords:** dendritic cells, inflammatory monocytes, macrophages, notch signaling

## Abstract

The clearance of the pathogenic fungus, *Histoplasma capsulatum*, requires cooperation between innate and adaptive immunity. Since this organism is inhaled, lung macrophages and dendritic cells (DCs) are the first lines of defense. Moreover, DCs act as APCs to drive the education of type 1 Th cells to produce IFNγ, which contributes to the final elimination of *H. capsulatum*. In this study, we explored the importance of Notch signaling in host defenses using a mouse model of pulmonary histoplasmosis. We found up‐regulation of Notch ligands (NLs) and Notch receptors (NRs) on phagocytes and IFNγ^+^ CD4^+^ T cells upon infection in lungs and lymph nodes. To ascertain the influence of Notch on the course of infection, we used a gamma‐secretase inhibitor (GSI), LY‐411,575, which inhibits NR downstream signaling. This compound impaired fungal clearance when given at the time of infection or 7 days after infection. However, GSI did not impact fungal clearance in mice with preexisting immunity. The dampened host defenses were associated with reduced differentiation and maturation of monocyte‐derived DCs and elevatmonocyte‐derived macrophage and alveolar macrophage polarization to M2. Our study reveals the critical nature of Notch signaling in maintaining control of this infectious agent.

## Graphical Abstract

Notch signaling dictates the function of dendritic cells and monocytes/macrophages in fungal infection.

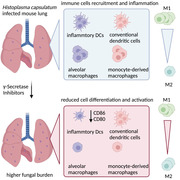

**Abbreviations**
(AMOs): alveolar macrophage
(BMDC): bone marrow‐derived DC
(BMDMs): bone marrow‐derived macrophages
(cDCs): conventional DCs
(DLL4): Delta‐like4
(DC): dendritic cell
(FMO): Fluorescent Minus One
(GSI): gamma secretase inhibitor
(Inf DCs/MΦs): inflammatory DCs/macrophages
(iMonos): inflammatory monocytes
(i.n.): intranasally
(i.p.): intraperitoneally
(KLF2): Krüppel‐like factor 2
(MFI): mean fluorescence intensity
(Mo‐AMs): monocyte‐derived macrophage
(MOI): multiplicity of infection
(NICD): Notch intracellular domain
(NL): Notch ligand
(NR): Notch receptor
(PI): postinfection
(RBPJ): recombination signal binding protein for immunoglobulin Jκ region
(Retnlα): resistin‐like α
(DAPT): *Tert*‐butyl (*S*)‐{(2*S*)‐2‐[2‐(3,5‐difluorophenyl) acetamido] propanamido} phenylacetate

## 1 INTRODUCTION

*Histoplasma capsulatum* is an intracellular dimorphic fungus found in the soil environment enriched by bird and bat droppings.^1^ Histoplasmosis is the disease caused by this fungus, which is endemic to the Mississippi River and Ohio River valleys in the United States. Through evolution, this organism has adapted to survive in a mammalian host. Upon inhalation, it converts from the saprobic phase to the pathogenic yeast phase in lungs. For immunocompetent patients, *H. capsulatum* infection typically causes self‐limited illness. In immunocompromised patients, especially in those with impaired cell‐mediated immunity, a life‐threatening disease may develop.^2^ Both innate and adaptive immunity are required in clearing *H. capsulatum* infection.

Yeast cells invade mononuclear phagocytes to avoid damage from the extracellular environment such as surfactant proteins in the human host.^3^ Upon settling in lungs, alveolar macrophages (AMOs) ingest the organism and serve as a nidus for conversion to yeast cells. Subsequently, these fungal elements disseminate to multiple tissues where they infect other phagocytes.^4^ The fate of the organism in phagocytes varies with the population; polymorphonuclear leukocytes are fungistatic, monocytes/macrophages allow fungal growth, and dendritic cells (DCs) retard the growth of the fungus.^5^, ^6^, ^7^ Optimal clearance of *H. capsulatum* requires activation of the adaptive immune response. The initiation of T cell‐mediated immunity relies on various DC populations, including monocyte‐derived DCs, CD11b^+^ conventional DCs (cDCs) and CD11b^+^ cDCs. These cells present fungal antigens to T cells and promote induction of type 1 Th1 cells.^8^ IFNγ from these T cells endows monocytes and macrophages with the capacity to limit the growth of this fungus and hasten its elimination.^9^

Notch signaling is an evolutionarily conserved pathway present in most animals.^10^ The key role of the Notch pathway in immune regulation has acquired more attention in recent decades since a group reported chromosomal translocation of the human *Notch1* gene in T cell lymphoblastic cells.^11^ There are 5 Notch ligands, including Jagged1 and Jagged2, Delta‐like1 (DLL1), DLL3, and 4. The 4 Notch receptors, Notch1, 2, 3, and 4, bind to NLs and are cleaved by multiple proteases in a stepwise fashion, starting with a distintegrin and metalloprotease and finishing with γ‐secretase, to release the Notch intracellular domain (NICD) from the cell membrane to cytoplasm.^12^, ^13^ NICD enters the nucleus along with the transcription factor recombination signal binding protein for immunoglobulin Jκ region (RBPJ) and other coactivators to initiate gene expression.^14^ Notch signaling regulates innate immune responses expressed by monocytes and macrophages and DCs. Notch activation inhibits alternatively activated (M2) macrophage polarization through repressing the signal regulatory protein α, while classically activated (M1) macrophages are essential in clearing *H. capsulatum* infection.^15^, ^16^ Notch controls the differentiation and homeostasis of monocyte‐derived DCs and is an important bridge between innate cells and T cells. ^17^, ^18^ Two theories have emerged regarding Notch signaling and T cell differentiation. One is the “instructive model” in which the specific binding of NLs on APCs to NR on T cells induces Th1 or Th2 polarization. The second is the “unbiased amplifier model” in which Notch functions as a general amplifier and facilitates Th1, Th2, Th9, Th17, and T reg differentiation.

In this manuscript, we probed the importance of Notch signaling in controlling *H. capsulatum* infection. We demonstrated the dynamic expression of NLs and NRs on phagocytes and Th1 cells in lungs and lymph nodes (LNs). To uncover the contribution of Notch, we utilized a γ‐secretase inhibitor (GSI) to prevent the release of NICD and NR downstream gene expression in *H. capsulatum* infected C57BL/6J mice. Treatment with a GSI dampened fungal clearance in primary but not secondary infection. Loss of control was observed during the innate and adaptive immune response phase. The higher fungal burden was associated with a diminished number of monocyte‐derived DCs, which we refer to as inflammatory DCs/macrophages (Inf DCs/MΦs) in this paper due to the constant changing and evolving definitions for monocyte‐derived DCs, a higher number of inflammatory monocytes, and an elevated proportion of M2 macrophages in lungs. The generation of cytokines that promote protective immunity was unaltered. Mechanistically, GSI directly reduced Inf DCs/MΦs differentiation and activation. Thus, Notch signaling is essential in regulating monocyte/macrophages and Inf DCs/MΦs’ response but not T cells in regulating *H. capsulatum* infection.

## 2 MATERIALS AND METHODS

### 2.1 Mice

C57BL/6 mice were housed in pathogen‐free conditions with Laboratory Animal Medical Services under the University of Cincinnati Animal Care and Use Program and the Institutional Animal Care and Use Committee regulations. C57BL/6 mice were purchased from Jackson Laboratories (Bar Harbor, ME, USA) and used at 9–16 week of age. All animal experiments were performed in accordance with the Animal Welfare Act guidelines of the National Institutes of Health, and all protocols were approved by the Institutional Animal Care and Use Committees of the University of Cincinnati.

### 2.2 *H. capsulatum* infection and fungal burden quantitation

*H. capsulatum* yeast strain G217B and G217B GFP^+^ were cultured in HAMs F‐12 medium (pH = 7.5) supplemented with glucose (18.2 g/L), glutamic acid (1 g/L), cysteine (0.17 g/L), HEPEs (6 g/L), and grown in shaking incubator at 200 rpm for 72 h at 37°C. Mice were infected intranasally (i.n.) with 2 × 10^6^ yeast cells suspended in ∼35 μl of HBSS. The lungs were homogenized in a Tenbroeck tissue grinder, serially diluted in HBSS, and 50 μl was plated onto Mycosel‐agar™ plates containing 5% sheep blood and 5% glucose to perform fungal burden. *H. capsulatum* CFUs were enumerated after incubation at 37°C for 7–10 days.

### 2.3 GSI treatment

Different types of GSI treatment were used including 7 mg/kg LY‐411,575 (Cayman Chemical, Ann Arbor, MI, USA) and 5 mg/kg DAPT (*tert*‐Butyl (*S*)‐{(2*S*)‐2‐[2‐(3,5‐difluorophenyl) acetamido] propanamido} phenylacetate; Cayman Chemical). The GSI was reconstituted in DMSO and injected into mice intraperitoneally (i.p.) with 200 μl PBS or directly added into cell cultures in vitro. An equivalent concentration of DMSO suspended in 200 μl PBS was used as control. Mice were treated with GSI daily starting a day in advance of *H. capsulatum* infection until harvest day.

### 2.4 Preparation of lung and LN leukocytes

Lungs were homogenized in C tubes with gentleMACS Dissociator (Miltenyi, Auburn, CA, USA) according to manufacturer's protocol and digested with 2 mg/ml Collagenase D (Sigma–Aldrich, St. Louis, MO, USA) and 80 U/ml DNase I (Sigma–Aldrich) for 30 min at 37°C. Cells were filtered with a 70 μm mesh cell strainer and suspended in HBSS with 1 mM EDTA. Single cell suspensions of mediastinal LNs were teased apart with plunger flange and passed through a 70 μm mesh cell strainer.

### 2.5 Bone marrow‐derived DCs and bone marrow‐derived macrophages culture

Femurs and tibiae were excised from C57BL/6 mice and flushed with PBS through a 25G needle. Cells were centrifuged at 400×*g* and suspended in 5 ml of ammonium‐chloride‐potassium red blood cell lysing buffer (150 mM NH_4_Cl, 10 mM KHCO_3_, 0.1 mM Na_2_EDTA) for 5 min at room temperature. After 2 washes with PBS, cells were resuspended in complete RPMI medium (RPMI with 10% FBS, 1% gentamicin, and 5 × 10^−5^ M 2‐mercaptoethanol) and pipetted through 70 μm strainer to acquire single cell suspensions. BM cells were added into 300 cm2 culture flasks with 50 ml complete RPMI at 1 × 10^6^ cells/ml concentration and 10 ng/ml GM‐CSF. Cultures were incubated at 37°C with 5% CO_2_ for 7 days. On day 4, 50 ml fresh complete RPMI with 10 ng/ml GM‐CSF was added to cultures. On day 7, floating cells and loosely adherent cells were harvested and percolated through a CD11c positive selection column (Miltenyi) according to the manufacturer's protocol to acquire bone marrow‐derived DCs (BMDCs). The adherent cells were treated with 0.025% trypsin at 37°C with 5% CO_2_ for 10 min and then collected with a cell scraper to acquire bone marrow‐derived macrophages (BMDMs). Five hundred microliters of 10^6^ BMDCs were seeded in nontissue‐treated 24‐well plates for 2 h before infection or stimulation. BMDCs were infected with a multiplicity of infection (MOI) of 2 yeast cells per BMDC with or without Syk inhibitor, BAY 61–3606 (Cayman Chemical) or LY‐411,575. Two hundred microliters of 2 × 10^5^ BMDMs were seeded on tissue‐treated 96‐well plate for 18 h followed by 2 h DMSO or GSI treatment. BMDMs then were infected with an MOI of 1 for 48 h.

### 2.6 Cell surface staining and intracellular staining

Lung and LN leukocytes were lysed in 5 ml of red blood cells lysis buffer for 5 min at room temperature. One million cells were incubated with anti‐CD16/32 (Leinco, St. Louis, MO, USA) followed by incubation with the following monoclonal antibodies in 100 μl FACs buffer for 20 min at 4°C. BD Biosciences (NJ, USA) Abs: CD11c PE‐Cy7 (clone: HL3), CD11b Percp‐Cy5.5 (clone: M1/70), Ly6C PE (clone: AL‐21), CD4 APC‐Cy7 (clone: GK1.5), and CD62L PE (clone: MEL‐14). Biolegend (San Diego, CA, USA) Abs: CD64 BV605 (clone: X54‐5/7.1), MHC class II Alexa Fluor 700 (MHC II, clone: M5/114.15.2), CD206 PE (clone: C068C2), CD86 BV510 (clone: GL‐1), CD40 PE (clone: FGK45), CD80 BV510 (clone: 16‐10A1), Delta‐like 4 PE (DLL4, clone: HMD4‐1), CD14 APC (clone: SA14‐2), CD45 BV510 and F1TC(clone: 30‐F11), CD3 Alexa Fluor 700 (clone: 17A2), Notch 1 APC (clone: 22E5), Notch 2 APC (clone: HMN2‐35), Notch 3 Alexa Fluor 647 (clone: HMN3‐133), Jagged 1 PE (clone: HMJ1‐29), PD1 BV605 (clone: 29F.1A12), and Streptavidin Alexa Fluor 647. Invitrogen (Carlsbad, CA, USA) Abs: CD24 APC (clone: M1/69) and inducible T‐cell costimulatory F1TC (ICOS, clone: 7E.17G9). Miltenyi Biotec Abs: Jagged2 Biotin.

Cells were stained with Fixable Viability Dye eFluor 450 (eBioscience, San Diego, CA, USA) in 100 μl PBS for 20 min at 4°C. In the end, cells were fixed with 2% PFA 10 min at room temperature. Intracellular stimulation was performed with 1 μg/ml ionomycin (Calbiochem, St. Louis, MO, USA) and 200 ng/ml phorbol myristate acetate (Millipore Sigma, St. Louis, MO, USA) for 4 h followed by the addition of 3 μg/ml brefeldin A (eBioscience) for 2 h in 96‐well U bottom plates. For intracellular staining, cells were fixed and permeabilized with BD Cytofix/Cytoperm kit according to the manufacturer's recommendation. Intracellular staining was performed with monoclonal antibody to IL‐4 PE (clone:11B11; Invitrogen) and IFNy F1TC (clone: XMG 1.2; BD) in 50 μl FACs buffer for 45 min at 4°C. All flow cytometric data were acquired using Canto 3 (BD) maintained by the Research Flow Cytometry Core at Cincinnati Children's Hospital Medical Center.

We identified lung phagocytes with following markers: monocytes (MHCII^−^ CD11b^+^CD64^+^); AMOs (MHCII^+/−^ CD11c^+^CD11b^−^CD64^+^); interstitial macrophages (MHCII^+/−^ CD11c^−^CD11b^+^CD24^+^); Inf DCs/MΦs (MHCII^+^CD11c^+^CD11b^+^ CD64^+^); CD11b^+^ cDCs (MHCII^+^CD11c^+^CD24^+^ CD64^−^CD11b^+^); CD11b^−^ cDCs (MHCII^+^CD11c^+^CD24^+^ CD64^−^CD11b^−^); CD4^+^ T cell (CD3^+^CD4^+^). All gating was made according to Fluorescent Minus One (FMO).

### 2.7 Isolation of F4/80^+^ lung macrophages

At day 5 PI, lungs were homogenized via gentleMACS dissociator in 5 ml complete RPMI with 2 mg/ml collagenase D and 40 U DNase I (Sigma–Aldrich) for 30 min at 37°C. Erythrocytes were lysed with an ammonium–chloride–potassium buffer. Cells were filtered through a 70 μm nylon mesh, washed, and counted. Anti‐F4/80 MicroBeads were used for the positive selection of F4/80^+^ cells (Miltenyi Biotec).

### 2.8 Quantitative real‐time PCR of cDNA synthesis

The cells were acquired from lung homogenate or cultured BM. Total RNA was purified with PureLink RNA Mini Kit (Invitrogen), and cDNA was synthesized with Oligo (dT) Primers and reverse transcriptase system (Promega, Madison, WI, USA) according to the manufacturer's instructions. The analysis of cytokine transcription was conducted with ABI Prism 7500 (Applied Biosystems, MA, USA), TaqMan® fast PCR master mixture, and primers (Applied Biosystems). Hypoxanthine Phosphoribosyltransferase 1 was used as the housekeeping gene and computation of fold change is calculated with delta‐delta Ct method with F4/80^+^ cells in DMSO‐treated mice lungs or uninfected BM‐derived cells as the baseline. The amplification was initiated at 50°C for 2 min and 95°C for 10 min. The amplification cycles were set up at 95°C for 15 s and 60°C for 1 min.

### 2.9 Statistical analysis

Data were analyzed using unpaired Student's *t*‐test, one‐way analysis of variance (ANOVA), or two‐way ANOVA with the confidential level of 95%.

## 3 RESULTS

### 3.1 Evolution of expression of NLs and NRs on phagocytes in the infected lung and mediastinal LN

**FIGURE 1:**
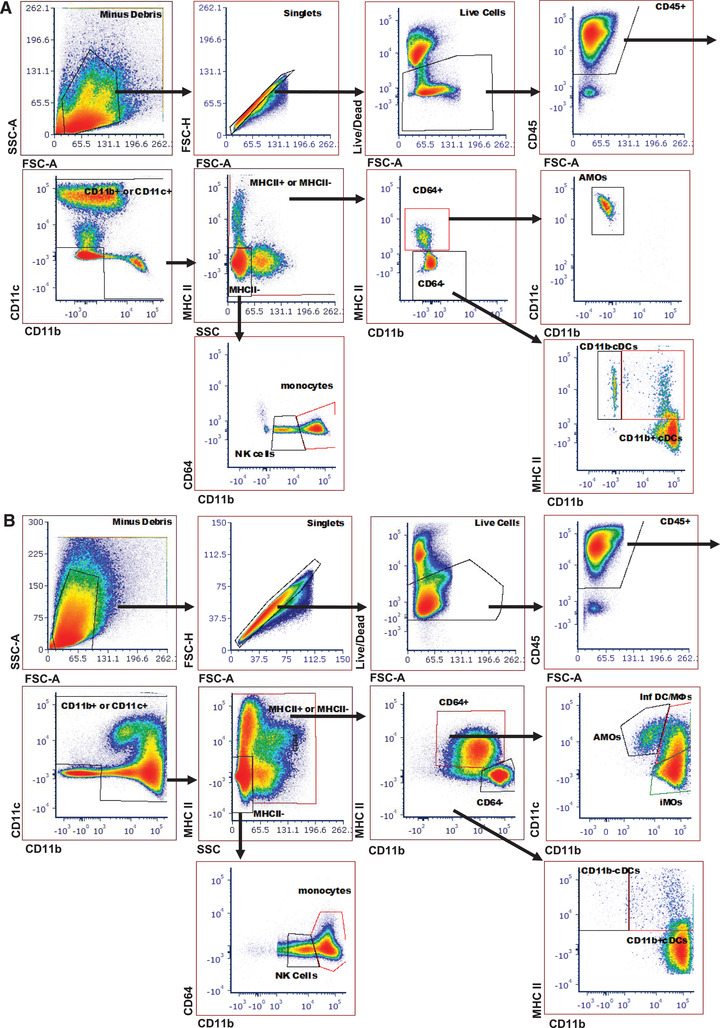
(A and B) Flow cytometry gating for various cell populations in infected lungs. Representative figures of lung leukocytes in naïve mice (A) and at day 4 PI mice (B)

A temporal analysis of NRs and NLs on immune cells has not been previously examined in *H. capsulatum* infection. Hence, we undertook a series of experiments to ascertain how this fungal infection shaped the display of NLs and NRs. We began with a flow cytometric analysis of phagocytes before infection (D0). Our study encompassed D4 postinfection (PI) as representative of innate immunity and D7 PI as illustrative of adaptive immunity. The gating strategy, which was adapted from a complete flow cytometric analysis of mouse lung, is shown in Figure 1.^19^ We identified Inf DCs/MΦs, CD11b^+^conventional DCs (CD11b^+^cDCs), CD11b^−^cDCs, AMOs, and monocytes, all of which are essential for regulating *H. capsulatum* infection in lungs.^7^, ^20^, ^21^, ^22^ We chose to focus on the NL, DLL4, which is important in Th1‐mediated response in lungs of mice with mycobacterial infection.^23^ We also analyzed the Th2‐related NL, Jagged2, which is reported to be crucial in Th2 development in lungs of *H. capsulatum* infected mice lacking the transcription factor Krüppel‐like factor 2 (KLF2).^24^ The NRs, Notch1, and 2 are critical in various lung diseases, hence we surveyed their expression on phagocytes as well.^25^

**FIGURE 2:**
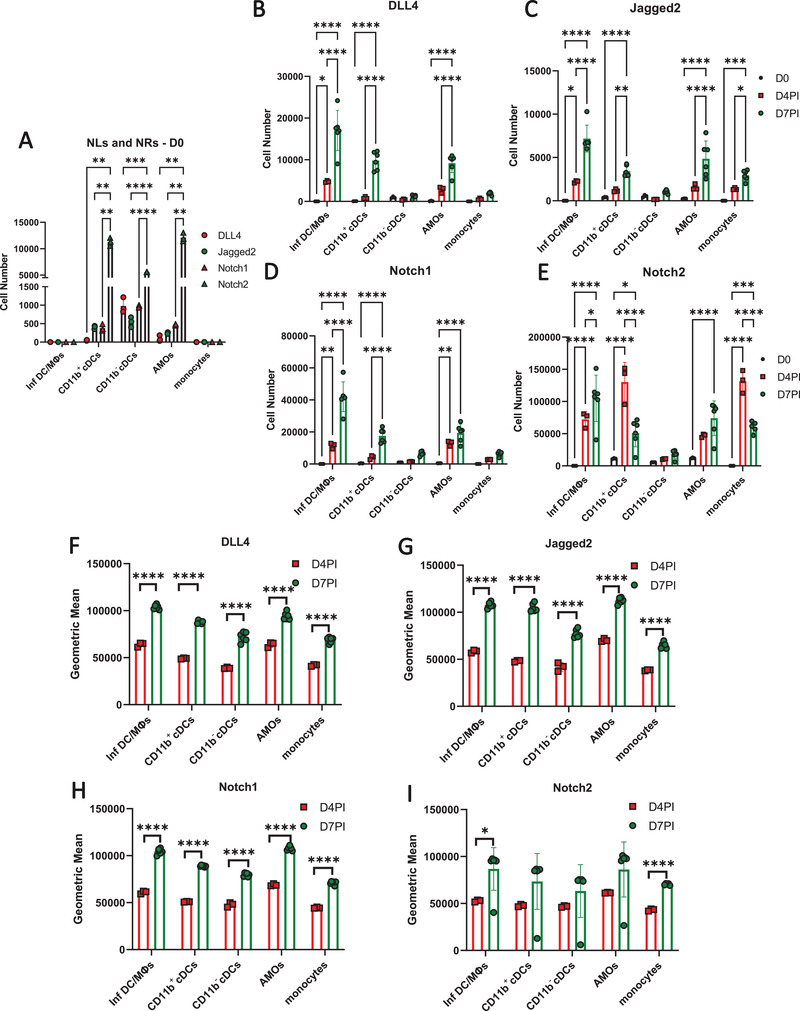
NL and NR expression on phagocytes in lungs of mice infected with *H. capsulatum*. Mice were infected with 2 × 10^6^ *H. capsulatum* (i.n.) and lungs were harvested prior to infection (day 0), and days 4 and 7 PI. Cell populations were analyzed with flow cytometry. (A) The number of NLs and NRs expressing phagocytes in lungs at D0 (*n* = 3). (B–E) The number of phagocytes expressing DLL4 (B), Jagged2 (C), Notch1 (D), and Notch 2 (E) in lungs at days 0, 4, and 7 PI (*n* = 3–6). Two‐way ANOVA followed by Tukey multiple comparison test. (F–I) The MFI of DLL4 (F), Jagged2 (G), Notch1 (H), and Notch2 (I) on phagocytes in lungs at days 4 and 7 PI (*n* = 3–6). Unpaired Welch's Student *t*‐test. Data represent the mean ± sd. ns: *p *> 0.05, *p < 0.05, **p < 0.01, ***p < 0.001, ****p < 0.0001

In lungs of uninfected mice, there were a small number of cells exhibiting NLs and NRs while most phagocytes manifested Notch2 (Figure 2(A)). No Notch2^+^ Inf DCs/MΦs were observed since there was no monocyte differentiation in lungs before infection. On days 4 and 7 of infection, a higher number of all lung phagocytes, excluding monocytes, were DLL4^+^ rather than Jagged2^+^ (Figures 2(B) and 2(C)). The number of monocytes bearing DLL4 and Jagged2 was similar. Regarding NRs, the largest number of Notch1^+^ cells were Inf DCs/MΦs and AMOs. In contrast, Notch2^+^ CD11b^+^ cDCs and monocytes were prevalent at day 4 PI, while the number of Notch2^+^ Inf DCs/MΦs and AMOs increased by day 7 PI (Figures 2(D) and 2(E)). The representative plots of the percentage of NLs and NRs expressing cells among Inf DCs/MΦs, CD11b^+^ cDCs, CD11b^−^ cDCs, AMOs, or monocytes in naïve lungs and days 4 as well as 7 PI lungs are shown in Figures [Link], [Link], [Link], [Link].

**FIGURE 3:**
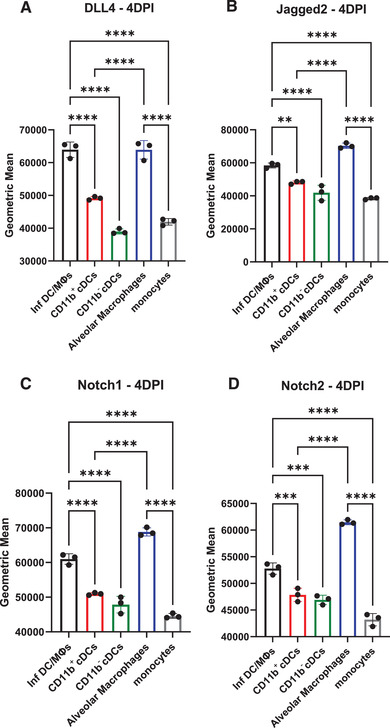
The MFI of NLs and NRs on various phagocytes in mice lungs. Mice were infected with 2 × 10^6^ *H. capsulatum* (i.n.) and lungs were harvested at day 4 PI. Various cell populations were analyzed with flow cytometry. (A–D) The MFI of DLL4 (A), Jagged2 (B), Notch1 (C), and Notch2 (D) on various phagocytes in lungs at day 4 PI (*n* = 3). One‐way ANOVA followed by Tukey multiple comparison test

We asked in this series of experiments whether there was a shift in the display of NLs and NRs on phagocytes as the infection progressed. On day 7 of infection, an elevated number of phagocytes bore NLs and NRs, other than Notch2, as compared with day 4 PI in lungs (Figures 2(B)–2(E)). Expression of NLs and NRs as determined by mean fluorescence intensity (MFI, geometric mean), progressively increased and peaked at day 7 PI in lungs (Figures 2(F)–2(I)). When we analyzed all phagocyte populations, we observed fewer CD11b^−^ cDCs exhibiting NLs and NRs in lungs (Figures 2(B)–2(E)). By day 7 PI, Inf DCs/MΦs became the major cell population manifesting NLs and NRs in lungs. (Figures 2(B)–2(E)). The MFI of NLs and NRs on Inf DCs/MΦs and AMOs exceeded that of other phagocytes in lungs (Figures 3(A)–3(D)).

**FIGURE 4:**
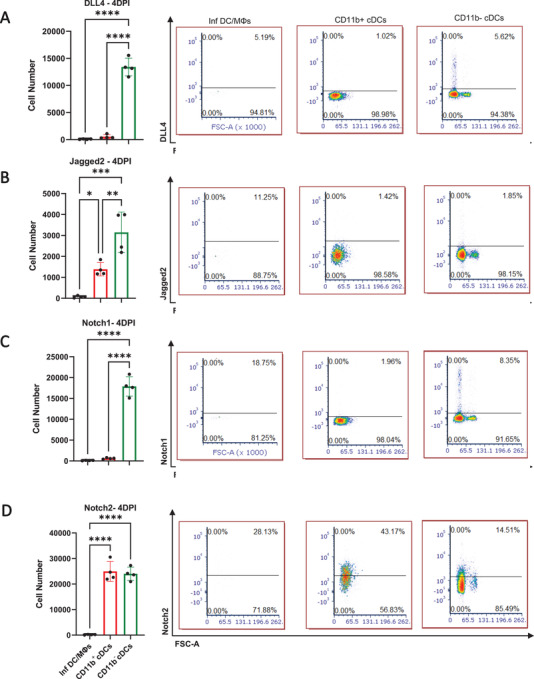
NL and NR on phagocytes in mediastinal LNs of *H. capsulatum‐*infected mice. Mice were infected with 2 × 10^6^ *H. capsulatum* (i.n.) and LNs were harvested at day 4 PI. Cell populations were analyzed with flow cytometry. (A–D) The number of various Phagocytes expressing DLL4 (A), Jagged2 (B), Notch1 (C), and Notch2 (D) in LNs at day 4 PI (*n* = 4). Representative plots of the percentage of NL and NR expressing cells among Inf DCs/MΦs, CD11b^+^ cDCs, and CD11b^−^ cDCs are shown. One‐way ANOVA followed by Tukey multiple comparison test. Data represent the mean ± sd. ns: *p* > 0.05, *p < 0.05, **p < 0.01, ***p < 0.001, ****p < 0.0001

In mediastinal LNs of uninfected mice, we were unable to detect NLs and NRs on phagocytes due to a paucity of cells. During infection, the number of NL^+^ or NR^+^ CD11b^−^ cDCs, which are critical in antigen presentation and T cell polarization, were more prominent in LNs than in lungs (Figures 4(A)–4(D)). We detected the largest number of DLL4^+^, Jagged2^+^, and Notch 1^+^ CD11b^−^ cDCs, whereas the number of Notch2^+^ CD11b^+^ and CD11b^−^ cDCs at day 4 PI were similar (Figures 4(A)–4(D)). Meanwhile, the number of DLL4^+^ CD11b^−^cDCs was higher than Jagged2^+^ CD11b^−^cDCs at day 4 PI (Figures 4(A) and 4(B)). Taken together, these data demonstrate that infection with this intracellular pathogen induced a dynamic change in NL and NR expression during the acute phase of the infection in lungs and LNs.

### 3.2 The expression of Notch on phagocytes is regulated through phagocytosis and Syk signaling

**FIGURE 5:**
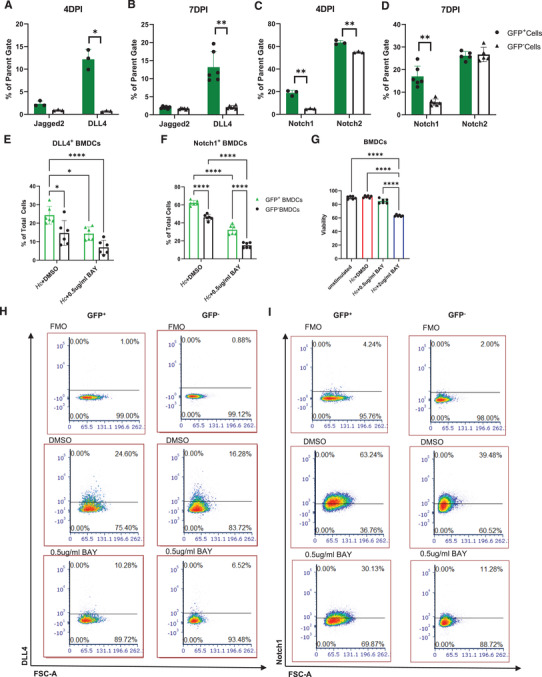
NL and NR expression on infected and uninfected APCs and dependence on Syk signaling. Mice were infected with 2 × 10^6^ GFP^+^ *H. capsulatum* (i.n.) and lungs were harvested at days 4 and 7 PI. Cell populations were analyzed with flow cytometry. (A and B) The percentage of Jagged2 or DLL4 expressing cells among GFP^+^ or GFP^−^ DCs (CD11c^+^CD11b^+^MHCII^+^) in lungs at day 4 (A) or day 7 (B) PI (*n* = 3–6). (C and D) The percentage of Notch1 or Notch2 expressing cells among GFP^+^ or GFP^−^ DCs (CD11c^+^CD11b^+^MHCII^+^) in lungs at day 4 (C) or day 7 (D) PI (*n* = 5–6). Unpaired Welch's Student *t*‐test. GMCSF differentiated BMDCs were infected with 2 MOI of *H. capsulatum* and treated either DMSO or BAY 61–3606 for 16 h. (E and F) The percentage of DLL4^+^ (E) and Notch1^+^ (F) cells among *H. capsulatum* GFP^+^ or GFP^−^ BMDCs population (*n* = 6). Two‐way ANOVA followed by Tukey multiple comparison test. (G) The viability of BMDCs (*n* = 6). One‐way ANOVA followed by Tukey multiple comparison test. (H and I) Representative plots of the percentage of DLL4 expressing (H) and Notch1 expressing (I) cells among *H. capsulatum* GFP^+^ or GFP^−^ BMDCs population (n = 6). Data represent the mean results ± . ns: *p* > 0.05, *p < 0.05, **p < 0.01, ***p < 0.001, ****p < 0.0001

The above experiments do not separate NL and NR display on uninfected from infected cells in the lung environment. To determine if the invasion of phagocytes dictated the increase in NLs and/or NRs, we infected mice with GFP^+^ *H. capsulatum* yeast cells. GFP^+^ DCs (MHCII^+^CD11b^+^ CD11c^+^) manifested a higher percentage of DLL4 but not Jagged2, as compared with GFP^−^ DCs at days 4 and 7 PI (Figures 5(A) and 5(B)). Meanwhile, a higher percentage of Notch1^+^ DCs was observed in the GFP^+^ DCs compared with GFP^−^ DCs at days 4 and 7 PI (Figures 5(C) and 5(D)). To ascertain if this finding was merely a consequence of infection or the environment induced by infection, we examined NL and NR expression on BMDCs. These in vitro results documented that fungal invasion alone altered the expression of NLs and NRs on BMDCs independent of the lung environment (Figures 5(E) and 5(F)).

Syk signaling influences the response to fungal infection in DCs and is associated with Notch signaling.^26^, ^27^ Hence, we applied a syk inhibitor, BAY 61–3606, to infected BMDCs. Based on cell toxicity studies (Figure 5(G)), we chose to use 0.5 μg/ml. We observed that inhibition of syk signaling reduced NL and NR expression both on GFP^+^ BMDCs and GFP^−^ BMDCs (Figures 5(E) and 5(F)). The representative plots of the percentage of DLL4 and Notch1 expressing cells among *H. capsulatum* GFP^+^ or GFP^−^ BMDCs population are shown in Figures 5(H) and 5(I). Therefore, syk is needed for appropriate Notch signaling expression on BMDCs.

### 3.3 NRs expression on CD4^+^ T cells in lungs and mediastinal LNs

**FIGURE 6:**
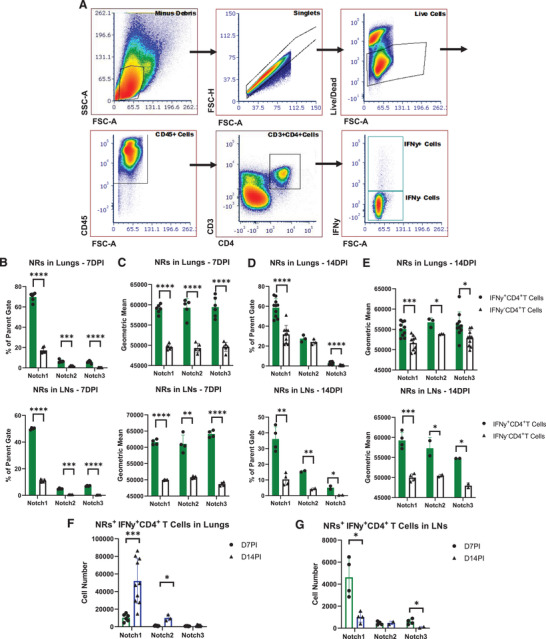
NL and NR on CD4^+^ T Cells in lungs during the adaptive immune response phase. Mice were infected with 2 × 10^6^ *H. capsulatum* (i.n.) and lungs were harvested at days 7 and 14 PI. (A) Flow cytometry gating for IFNγ^+^ or IFNγ^−^ CD4^+^ T cells in infected mice lungs. Representative figures of lung leukocytes in mice at day 7 PI. (B and C) The percentage (B) and the MFI (C) of NR^+^ cells among IFNγ^+^ CD4^+^ T cells or IFNγ^−^CD4^+^ T cells in lungs and LNs at day 7 PI (*n* = 4–6). (D and E) The percentage (D) and the MFI (E) of NR^+^ cells in IFNγ^+^ CD4^+^ T cells and IFNγ^−^CD4^+^ T cells in lungs and LNs at day 14 PI (*n* = 4–10). (F and G). The number of NR expressing IFNγ^+^CD4^+^ T cells in lungs (F) and in LNs (G) at days 7 and 14 PI (*n* = 4–10). Unpaired Welch's Student *t*‐test. Data represent the mean ± sd. ns: *p* > 0.05, *p < 0.05, **p < 0.01, ***p < 0.001, ****p < 0.0001

IFNγ^+^CD4^+^T cells are indispensable for eliminating *H. capsulatum*.^28^ There is strong evidence showing NR downstream signaling is key in peripheral T cells polarization.^29^ Hence, we examined the NR expression on IFNγ^+^CD4^+^T cells and IFNγ^−^CD4^+^T cells (the gating strategy is shown in Figure 6(A)). At day 7 PI, the percentage and MFI of NRs expressing IFNγ^+^CD4^+^T cells were elevated when compared with IFNγ^−^CD4^+^T cells in lungs and LNs (Figures 6(B) and 6(C)). The percentage of Notch1^+^ IFNγ^+^ CD4^+^ T cells was preponderant compared with other NR^+^ CD4^+^ cells. At day 14 PI, when the fungal burden declined, Notch1 maintained a greater frequency and MFI on IFNγ^+^CD4^+^T cells compared with IFNγ^−^CD4^+^T cells in lungs and LNs (Figures 6(D) and 6(E)). The representative plots of the percentage of NR expressing cells among IFNγ^+^ CD4^+^ T cells and IFNγ^−^ CD4^+^ T cells in lungs and LNs at days 7 and 14 PI are shown in Figure S5. Expression of Notch1 on IFNγ^+^CD4^+^T cells revealed an increase between days 7 and 14 of infection in lungs but not LNs (Figures 6(F) and 6(G)). Thus, Notch1 expression exhibited a strong association with IFNγ production, especially in lungs, due to the higher frequency and MFI compared with LNs.

### 3.4 Inhibition of Notch signaling impaired *H. capsulatum* clearance in lungs

**FIGURE 7:**
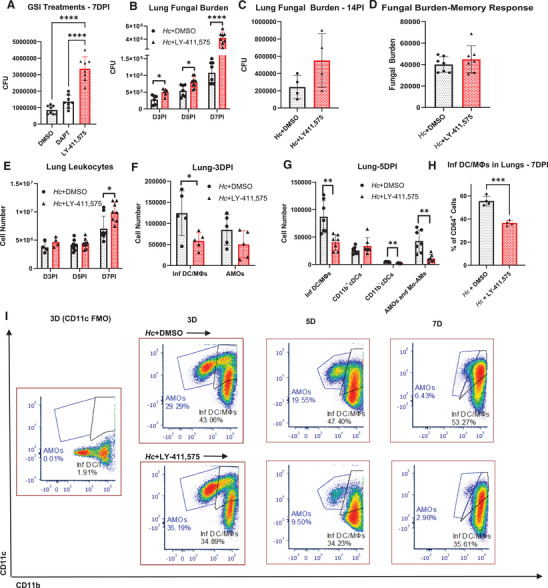
Effect of GSI treatment on fungal clearance and inflammation. Mice were treated with GSI (i.p.) 1 day before infection and continued daily until euthanasia. (A) Fungal burden in lungs of mice given vehicle control (DMSO), DAPT, or LY‐411,575 (*n* = 8). One‐way ANOVA followed by Tukey multiple comparison test. (B) Fungal burden in lungs with DMSO control or LY‐411,575 treatment at days 3, 5, and 7 PI (*n* = 6–8). Infected mice were administered vehicle control or LY‐411,575 from days 7 to 14 PI. (C) Fungal burden in lungs at day 14 PI (*n* = 4). (D) The fungal burden in lungs at day 7 PI after the mice were infected with *H. capsulatum* 8 week and reinfected for 7 days (*n* = 7). (E) The number of total lung leukocytes at days 3, 5, and 7 PI (*n* = 4–8). (F) The number of Inf DCs/MΦs and AMOs in the lung at day 3 PI (*n* = 5). (G) The number of phagocytes in the lung at day 5 PI (*n* = 7). (H) The percentage of Inf DCs/MΦs in the lung at day 7 PI (*n* = 4). Unpaired Welch's Student *t*‐test. (I) Representative plots of the percentage of AMOs and Inf DCs/MΦs among CD64^+^ cells in lungs at days 3, 5, and 7 PI (*n* = 4–7). Data represent the mean ± sd. ns: *p* > 0.05, *p < 0.05, **p < 0.01, ***p < 0.001, ****p < 0.0001

Based on the Notch expression profile on phagocytes and CD4^+^ T cells, we inquired whether Notch is essential for regulating *H. capsulatum* infection. The most vital step for Notch signaling downstream activation is the release of NICD through consecutive protease cleavage steps.^12^, ^13^ Among different protease inhibitors, GSI has been well studied for inhibiting NR cleavage.^30^ Previously we used GSI (LY‐411,575) in KLF2‐deficient mice, and we were able to reverse the effect of elevated NL expression. For our studies, we chose DAPT and LY‐411,575 although the latter has better specificity for NRs.^31^, ^32^ When we compared the 2 different GSIs, LY‐411,575 impacted fungal clearance more than DAPT (Figure 7(A)). LY‐411,575 caused a progressive increase in CFUs beginning as early as day 3 of infection and extending to day 7 (Figure 7(B)). We also asked if fungal clearance could be impacted after infection was established. Consequently, we treated infected mice with LY‐411,575 from days 7 to 14 PI daily. There was a higher fungal burden in LY‐411,575‐treated lungs at day 14 PI (Figure 7(C)). Next, we examined the possibility that GSI would alter the response of mice with preexisting immunity to rechallenge. Mice were infected with 2 × 10^6^ yeast cells (i.n.) and rechallenged 8 week later. Mice were treated with vehicle control or LY‐411,575. In contrast to primary infection, treatment with GSI did not alter the fungal burden (Figure 7(D)). Therefore, GSI blunted protective immunity in primary but not secondary infection.

We asked if the increased fungal burden in primary infection corresponded with poor inflammatory cell recruitment. The total number of lung leukocytes was similar between controls and GSI‐treated mice at days 3 and 5 PI. On day 7, the number of cells in LY‐411,575‐treated lungs exceeded that of controls (Figure 7(E)). Although total cell numbers were similar at days 3 and 5, we investigated if select phagocyte populations were altered in lungs of mice given LY‐411,575. We found fewer Inf DCs/MΦs at days 3 and 5 PI (Figures 7(F) and 7(G)). On day 7 PI, the number of total leukocytes was increased in LY‐411,575‐treated lungs; consequently, the total number of Inf DCs/MΦs was higher. However, the frequency of Inf DCs/MΦs among CD64^+^ cells was less in LY‐411,575‐treated lungs (Figure 7(H)). The representative plots of the percentage of AMOs and Inf DCs/MΦs among CD64^+^ cells in lungs at days 3, 5, and 7 PI are shown in Figure 7(I).

**FIGURE 8:**
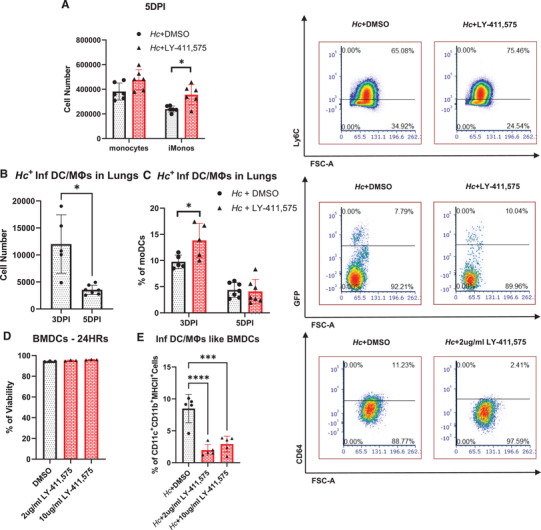
GSI treatment stimulated iMonos accumulation but reduced iMonos differentiation and Inf DCs/MΦs’ ability in killing *H. capsulatum*. Mice were treated with GSI (i.p.) 1 day before infection and continued daily until euthanasia. (A) The number of monocytes and iMonos in lungs at day 5 PI (*n* = 6). Representative plots of the percentage of iMonos among monocytes in lungs at day 5 PI are shown. Unpaired Welch's Student *t*‐test. Mice were infected with GFP^+^ *H. capsulatum* (i.n.). (B) The number of *H. capsulatum* ^+^ Inf DCs/MΦs in lungs at days 3 and 5 PI (*n* = 5–7). (C) The percentage of *H. capsulatum* ^+^ cells among Inf DCs/MΦs in lungs at days 3 and 5 PI (*n* = 5–7). Representative plots of the percentage of *H. capsulatum^+^* cells among Inf DCs/MΦs in lungs at day 3 PI are shown. Unpaired Welch's Student *t*‐test. BMDCs were infected with 2 MOI and treated either DMSO or LY‐411,575 for 24 h. (D) The viability of BMDCs (*n* = 3).(E). The percentage of CD64^+^ cells among BMDCs (*n* = 5). Representative plots of the percentage of CD64^+^ cells among BMDCs are shown. One‐way ANOVA followed by Tukey multiple comparison test. Data represent the mean ± sd. ns: *p* > 0.05, *p < 0.05, **p < 0.01, ***p < 0.001, ****p < 0.0001

In infected lungs, Inf DCs/MΦs are derived from recruited monocytes, especially inflammatory monocytes.^33^ We explored if the blunted number and frequency of Inf DCs/MΦs were a consequence of diminished monocyte recruitment. We chose day 5 PI since this is a time point that denotes the bridge between innate and adaptive immunity. Although Inf DCs/MΦs were diminished in GSI‐treated mice, the number of total monocytes was similar between the 2 groups and inflammatory monocytes (iMonos) were increased in LY‐411,575‐treated lungs (Figure 8(A)). These results suggest that the decrement in Inf DCs/MΦs was a result of impaired differentiation, not a paucity of inflammatory monocytes.

Initially, the CD11b^−^CD11c^+^CD64^+^ cell population denotes resident AMOs, and LY411,575 treatment did not alter their number at day 3 PI (Figure 7(F)). As inflammation progresses, monocytes differentiate into macrophages in lungs, and they are termed monocyte‐derived macrophages (Mo‐AMs, CD11c^+^CD11b^−/low^CD64^+^). They are presumed to replenish a reduced number of AMOs in lungs.^34^ The numbers of AMOs and Mo‐AMs were reduced in LY‐411,575‐treated lungs with *H. capsulatum* infection at day 5 PI (Figure 7(G)).

We determined if the ability of Inf DCs/MΦs to restrict the growth of *H. capsulatum* was damaged. The lower number of infected Inf DCs/MΦs, at day 5 PI compared with day 3, indicated Inf DCs/MΦs were capable of killing *H. capsulatum* in lungs (Figure 8(B)). There was a higher percentage of infected Inf DCs/MΦs at day 3 but not day 5 PI in LY‐411,575‐treated mice lungs, which revealed LY‐411,575 treatment undermined Inf DCs/MΦs growth restriction for only a short period (Figure 8(C)). To study if LY‐411,575 directly impeded Inf DCs/MΦs differentiation or was it due to an altered lung environment, we treated BMDCs with the GSI or vehicle and observed the differentiation of Inf DCs/MΦs. Upon exposure to LY‐411,575, fewer BMDCs manifested characteristics of Inf DCs/MΦs (CD64^+^) and the decrement was not caused by drug toxicity (Figures 8(D) and 8(E)). In conclusion, LY‐411,575 treatment compromised Inf DCs/MΦs differentiation and was correlated with higher inflammation in infected lungs.

### 3.5 LY‐411,575 treatment reduced expression of activation markers on Inf DCs/MΦs

**FIGURE 9:**
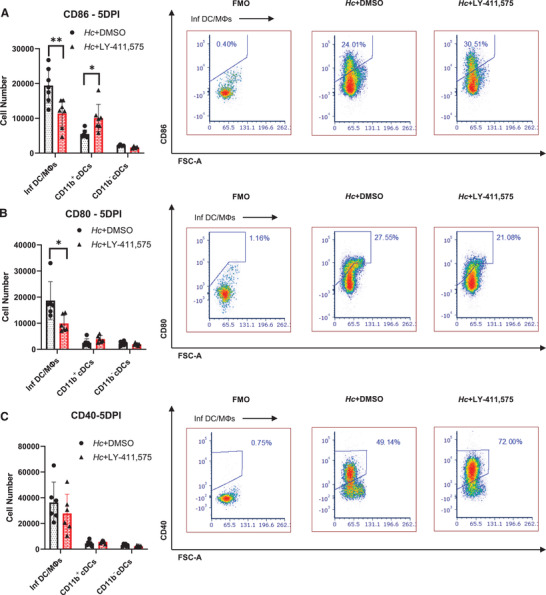
LY‐411,575 treatment impaired Inf DCs/MΦs but not cDCs activation. Mice were treated with LY‐411,575 (i.p.) 1 day before infection and continued administered daily until euthanasia. (A–C) The number of phagocytes expressing CD86 (A), CD80 (B), and CD40 (C) in lungs at day 5 PI (*n* = 4–7). Representative plots of the percentage of CD86, CD80, and CD40 expressing cells among Inf DC/MΦs in lungs at day 5 PI are shown. Unpaired Welch's Student *t*‐test. Data represent the mean ± sd. ns: *p* > 0.05, *p < 0.05, **p < 0.01, ***p < 0.001, ****p < 0.0001

DCs exhibit better growth inhibition of *H. capsulatum* than macrophages.^22^ They also limit the transformation of conidia to yeast form, which limits replication in the host.^7^ We demonstrated a lower number of infected Inf DCs/MΦs and lower frequency of Inf DCs/MΦs bearing intracellular fungus at day 5 PI compared with day 3 PI, which indicated Inf DCs/MΦs were capable of killing *H. capsulatum* in lungs (Figures 8(B) and 8(C)). We postulated that Inf DCs/MΦs are necessary for killing *H. capsulatum* in lungs, and the reduced number of Inf DCs/MΦs at D3 and D5 PI contributed to a higher fungal burden. Consequently, we investigated if Inf DCs/MΦ activation was impaired. CD40, CD80, and CD86, which are costimulatory molecules expressed on activated DCs, were examined.^35^, ^36^ There were fewer Inf DCs/MΦs bearing CD80 and CD86 but not CD40 in LY‐411,575‐treated lungs (Figures 9(A)–9(C)). Neither the number of CD80^+^ CD11b^−^cDCs nor CD11b^+^cDCs differed, but the number of CD86^+^ CD11b^+^ cDCs was higher in LY‐411,575‐treated lungs (Figures 9(A) and 9(B)). These data reveal that GSI decreased the activation phenotype of Inf DCs/MΦs but not cDCs.

### 3.6 LY‐411,575 treatment indirectly alters the polarization of inflammatory monocytes/macrophages

**FIGURE 10:**
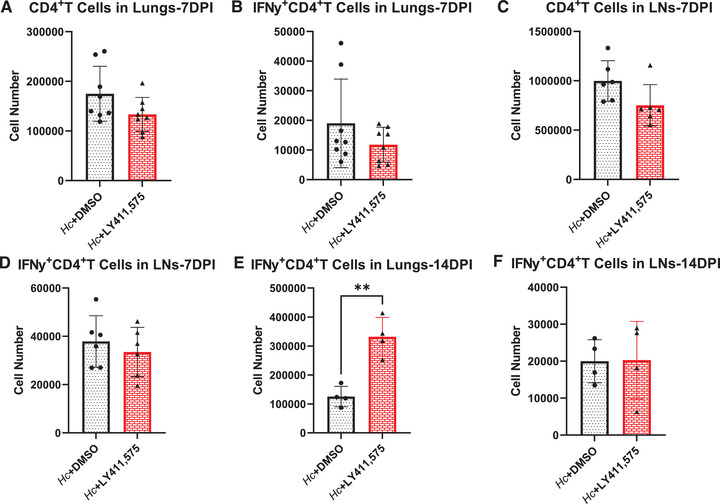
LY‐411,575 treatment and production of IFNγ. Mice were treated with LY‐411,575 (i.p.) beginning 1 day before infection and continued daily until euthanasia. (A and B) The number of CD4^+^ T (A) and IFNγ^+^ CD4^+^ T cells (B) in lungs at day 7 PI (*n* = 8). (C and D) The number of CD4^+^ T (C) and IFNγ^+^ CD4^+^ T (D) cells in LNs at day 7 PI (*n* = 6). Infected mice were administered vehicle control or LY‐411,575 from days 7 to 14 PI. (E and F) The number of IFNγ^+^ CD4^+^ T cells in lungs (E) and in LNs (F) at day 14 PI (*n* = 4). Unpaired Welch's Student *t*‐test. Data represent the mean ± sd. ns: *p* > 0.05, *p < 0.05, **p < 0.01, ***p < 0.001, ****p < 0.0001

Because our findings indicated that fungal clearance was impacted at day 7 PI, and there was a high association between Notch1 expression and IFNγ production, we sought to investigate if inhibition of NR downstream signaling would impact the production of IFNγ from CD4^+^ T cells. Flow cytometric analysis showed there was no difference in the number of CD4^+^ T cells or IFNγ^+^ CD4^+^ T cells in lungs and LNs at day 7PI (Figures 10(A)–10(D)). The number of IFNγ^+^ CD4^+^ T cells was also higher in LY‐411,575‐treated lungs but similar in LNs when the infection was established after day 7 PI (Figures 10(E) and 10(F)). Thus, Notch signaling is dispensable for CD4^+^ T cells to produce IFNγ.

**FIGURE 11:**
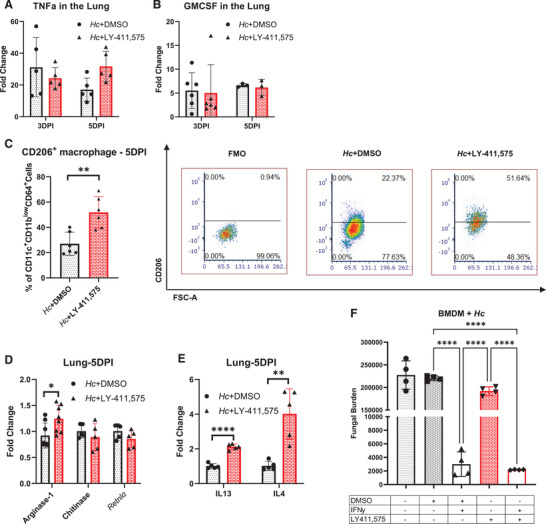
LY‐411, 575 impacted polarizations of monocyte/macrophages but not cytokine production and their responsiveness to IFNγ. (A and B) TNF‐α (A) and GM‐CSF (B) Fold change expression in lungs at days 3 and day 5 PI (*n* = 3–6). (C) The percentage of CD206^+^ cells among monocyte/macrophages in lungs at day 5 PI (*n* = 6). Representative plots of the percentage of CD206^+^ cells among macrophages are shown. (D) The fold change expression of Arginase‐1, Chitinase‐like molecules, and Retnlα in F4/80^+^ cells in lungs at day 5 PI (*n* = 5–8). (E) The fold change expression of IL‐13 and IL4 in lungs at day 5 PI (*n* = 5). Unpaired Welch's Student *t*‐test. (F) The fungal burden in BMDC culture with different combinations of DMSO control, IFNγ, or LY‐411,575 treatment after being infected for 48 h (*n* = 4). One‐way ANOVA followed by Tukey multiple comparison test. Data represent the mean ± sd. ns: *p* > 0.05, *p < 0.05, **p < 0.01, ***p < 0.001, ****p < 0.0001

To investigate what drives the higher fungal burden during the adaptive immune responses phase, we wondered if cytokines, other than IFNγ, which are necessary for protective immunity were diminished in LY‐411,575‐treated lungs. TNF‐α and GMCSF are essential for fungal clearance.^37^, ^38^ There were no difference between groups in TNF‐α or GM‐CSF cDNA expression in infected lungs at days 3 and 5 PI (Figures 11(A) and 11(B)).

Although IFNγ was not diminished, it was possible that cell responsiveness to it was dependent on Notch signaling. M1 inflammatory monocytes/macrophages respond to IFNγ to kill intracellular *H. capsulatum*.^39^ M2 inflammatory monocytes/macrophages prevent the clearance of *H. capsulatum* and manifest blunted responsiveness to IFNγ.^40^ First, we analyzed CD206, which is a characteristic of M2 macrophages.^41^ GSI treatment produced a higher proportion of CD206^+^ cells in lungs (Figure 11(C)). This finding led us to ask if macrophages exhibited other features consistent with an M2 phenotype. Arginase‐1, chitinase‐like molecules, and resistin‐like α (*Retnlα*) are identifiers of M2 macrophages in pulmonary fungal infection.^42^ We isolated F4/80^+^ macrophages from lungs of GSI‐treated and control mice. Lung macrophages from GSI‐treated mice manifested increased expression of arginase‐1, but not other genes (Figure 11(D)). During infection, macrophage polarization is regulated through cytokine production, in which IFNγ promotes M1 macrophages and IL4, as well as IL13, promotes M2 macrophages. ^39^, ^43^ We found elevated expression of IL4 and IL13 in GSI‐treated mice compared with control (Figure 11(E)). We also investigated the expression of IL4 by CD4^+^ T cells in GSI‐treated mice. The mean (±sd) number of IL4^+^ CD4^+^ T cells in GSI‐treated mice (31,724 ± 5422) exceeded (*p* = 0.0173) that of controls (13,852 ± 8685) at day 14 PI (*n* = 4). Next, we demonstrated that GSI did not directly impact macrophage responsiveness to IFNγ (Figure 11(F)). Therefore, inhibition of Notch advanced M2 polarization in *H. capsulatum* infected lungs indirectly.

## 4 DISCUSSION

We previously reported that a KLF2–Notch axis was key to restraining the differentiation and expansion of Th2 cells in histoplasmosis.^24^ In that study, KLF2‐deficient DCs overexpressed the NL, Jagged 2, which prompted the emergence of Th2 cells that negatively influenced the clearance of the fungus. In this endeavor, we explored whether Notch was pivotal for the handling of this fungal infection in wild‐type mice independent of persistent changes in KLF2. We found the major innate immune cell populations expressing NLs and NRs are Inf DCs/MΦ, Mo‐AMs, and AMOs in lungs, whereas a greater number of CD11b^−^ cDCs expressed NLs and NRs in LNs. *H. capsulatum*‐infected phagocytes expressed higher amounts of NLs and NRs than their uninfected counterparts. Also, DLL4^+^ phagocytes outnumbered those bearing Jagged2^+^. Regarding T cells, IFNγ^+^CD4^+^ T cells manifested elevated NRs and Notch1^+^IFNγ^+^CD4^+^ T cells were the predominant cell population among NR^+^IFNγ^+^CD4^+^ T cells. Enhanced expression of NRs and NLs prompted us to ask if Notch signaling contributed to host resistance. We applied a broad inhibitor, a GSI, LY‐411,575 to broach this question. This compound blunted fungal defenses during the innate and adaptive phases. The alterations associated with modified host defenses were reduced number of Inf DCs/MΦs, Mo‐AMs, and AMOs, increased number of inflammatory monocytes as well as increased frequency of M2 macrophages. We also discovered that the diminished number of Inf DCs/MΦs and Mo‐AMs were due to impaired monocyte differentiation rather than excessive cell death.

Fungal elimination in the lung was impaired as early as day 3 PI and continued to progress at day 5 PI in the GSI‐treated group. Before day 5, the innate immune response is the major contributor in limiting *H. capsulatum* infection. During this phase, neutrophils, macrophages, and DCs in lungs are the paramount cell populations that phagocytose these fungi.^21^ Neutrophils are fungistatic, DCs restrict *H. capsulatum* replication, and resident macrophages allow fungal growth until activated by IFNγ.^9^, ^20^ Likewise, Mo‐AMs, which are derived from monocytes, require activation by this cytokine to restrain intracellular growth.^9^, ^44^, ^45^

Recent studies have shown that Notch regulates the differentiation and activation of certain DC and macrophage populations.^46^, ^47^ Little is known about the influence of Notch signaling on the differentiation of Inf DCs/MΦs and Mo‐AMs specifically. Since GSI treatment did not impact monocyte recruitment, the most likely explanation is defective differentiation of Inf DCs/MΦs upon the interference of Notch signaling. This impact on differentiation was directly influenced by Notch signaling because we observed a diminished frequency of monocyte‐derived cells in GSI‐treated BMDCs with infection. Additional proof that Notch contributes to monocyte cell fate has been reported in cells exposed to a TLR7 stimulus.^48^ Another possible explanation for the reduced numbers of these cells is that GSI causes apoptosis.^49^ This reason is less likely because the number of total leukocytes was not diminished in GSI‐treated animals. GSI treatment did not reduce the viability of BMDCs and BMMs in vitro. Inhibition of Notch did not impact the differentiation and activation of cDCs in lungs, although Notch2 is central in cDC differentiation in other tissues such as the spleen.^50^

Differences in CFU were more pronounced at day 7 PI, which suggested that the failure of the innate immune system to control infection could not be rescued by the adaptive immune system. During the adaptive response, IFNγ is indispensable. The lack of this cytokine is associated with high mortality, and most mice die within the first 10 days of infection. IFNγ activates intracellular fungal killing by limiting the available metals in macrophage phagosomes.^9^, ^51^ Despite the diminished host resistance during the adaptive immune phase in GSI‐treated mice, IFNγ production by CD4^+^ T cells was not impaired in lungs and LNs. These results suggest that the Notch pathway was dispensable for Th1 cell polarization in *H. capsulatum* infection. Although the number of Inf DCs/MΦs and Mo‐AMs expressing costimulation markers CD80/CD86 was reduced in GSI‐treated animals, neither the number of cDCs nor their activation was altered. These results suggest that the former populations are more influential in host resistance than the latter.

In contrast to the primary infection, GSI treatment did not alter host control of *H. capsulatum* in a model of secondary histoplasmosis. Unlike the primary immune response, which relies on IFNγ, the secondary challenge is independent of this cytokine.^52^ TNFα, on the other hand, is crucial for survival in primary and secondary infection, but GSI did not alter the production of this cytokine in lungs.^53^ Therefore, it is more likely that GSI impacted the IFNγ responsiveness of macrophages, rather than their TNFα responsiveness. Although others reported that GSI controls memory CD4^+^ T cells survival in nitrophenyl immunized mice, our data indicated that GSI did not weaken memory immune response.^54^ We also investigated if Notch signaling plays a role in regulating adaptive immune responses in *H. capsulatum* infection since GSI compromised clearance when given at day 7 PI and continued until day 14 PI. Memory immune cells are formed as early as day 7 PI regarding *H. capsulatum* infection.^55^ This outcome implied that GSI treatment impacted an evolving, but not established memory response.

We identified 2 mechanisms by which GSI altered host defenses. First, a reduced number of AMOs and Mo‐AMs are present in lungs of GSI‐treated mice. Fewer cells translate into fewer IFNγ‐responsive cells. Second, we uncovered a higher frequency of M2 polarization of AMOs and Mo‐AMs in GSI‐treated lungs. These results are supported by prior work demonstrating that in lipopolysaccharides‐treated RAW264.7 macrophages, down‐regulation of Notch1 was associated with the up‐regulation of arginase‐1.^56^ As M2 are permissive for fungal growth, GSI dictated the polarization of AMOs and Mo‐AMs in lungs. This effect appears to be indirect since GSI did not alter IFNγ responsiveness by BMDM. Macrophages demonstrate an M2 profile in association with the up‐regulation of the type II cytokines, IL‐4, and IL‐13.^57^ IL‐4, in particular, is detrimental to the clearance of *H. capsulatum*.^40^ Therefore, one reasonable explanation is that M2 polarization was promoted by IL4 and IL13 production. Another explanation is that GSI can precondition inflammatory monocytes and macrophages to M2 during cell development.

One of the limitations of our experiments is that we only used a few cell surface markers for Inf DCs/MΦs (MHCII^+^CD11c^+^CD11b^+^ CD64^+^) to demonstrate various cell populations at the same time. Meanwhile, the definition for Inf DCs/MΦs is rapidly changing because of the evolution of high‐resolution single‐cell RNAseq. The definition of various cells, especially myeloid cells, is in flux as we learn more.

In summary, we demonstrated that Notch signaling is essential for regulating *H. capsulatum* infection. Inhibition with GSI impaired Inf DCs/MΦs and Mo‐AMs differentiation as well as activation and promoted macrophage polarization to M2. Our study provides an impetus for studying the Notch pathway in monocyte‐derived subpopulations. Clinically, GSI has been implemented in multiple clinical trials as a new candidate for cancer treatment because of its role in regulating NICD levels.^58^, ^59^ GSI also has been combined with CAR T cells to treat myeloma.^60^ Thus, our findings raise the possibility that the risk of fungal infection may be higher in those who receive this treatment. Our assertion may not be limited to *H. capsulatum* because monocyte‐derived cells and IFNγ responsiveness are proven to be crucial in multiple other fungal infections including *Aspergillus*, *Candida*, *Cryptococcus*, and *Blastomyces* species.

## AUTHORSHIP

S. H. designed and performed the experiments, analyzed the results, and wrote the manuscript. G. S. D. supervised the studies and assisted in manuscript preparation.

## DISCLOSURE

The authors declare no conflicts of interest.

## Supporting information

Figure S1. Representative plots of the percentage of DLL4 expressing cells among Inf DC/MΦs, CD11b+ cDCs, CD11b‐ cDCs, AMOs, or monocytes in naïve lungs and days 4 as well as 7 PI lungs (n=3‐6).No Inf DC/MΦs at D0

Figure S2. Representative plots of the percentage of Jagged2 expressing cells among Inf DC/MΦs, CD11b+ cDCs, CD11b‐ cDCs, AMOs, or monocytes in naïve lungs and days 4 as well as 7 PI lungs (n=3‐6).No Inf DC/MΦs at D0

Figure S3. Representative plots of the percentage of Notch1 expressing cells among Inf DC/MΦs, CD11b+ cDCs, CD11b‐ cDCs, AMOs, or monocytes in naïve lungs and days 4 as well as 7 PI lungs (n=3‐6). No Inf DC/MΦs at D0

Figure S4. Representative plots of the percentage of Notch2 expressing cells among Inf DC/MΦs, CD11b+ cDCs, CD11b‐ cDCs, AMOs, or monocytes in naïve lungs and days 4 as well as 7 PI lungs (n=3‐6).No Inf DC/MΦs at D0

Figure S5. Representative plots of the percentage of NR expressing cells among IFNγ+ CD4+ T cells and IFNγ‐ CD4+ T cells in lungs and LNs at days 7 and 14 PI (n=4‐10).
