# Supplementary material for: Notch regulates Histoplasma capsulatum clearance in mouse lungs during innate and adaptive immune response phases in primary infection
Source: J Leukoc Biol. 2022 May 23;112(5):1137–54. doi: 10.1002/JLB.4A1221-743R (PMC9613517; doi:10.1002/JLB.4A1221-743R)
Supplement: Supplementary file 4 — Figure S4. Representative plots of the percentage of Notch2 expressing cells among Inf DC/MΦs, CD11b+ cDCs, CD11b‐ cDCs, AMOs, or monocytes in naïve lungs and days 4 as well as 7 PI lungs (n=3‐6).No Inf DC/MΦs at D0 [file JLB-112-1137-s002.pdf]

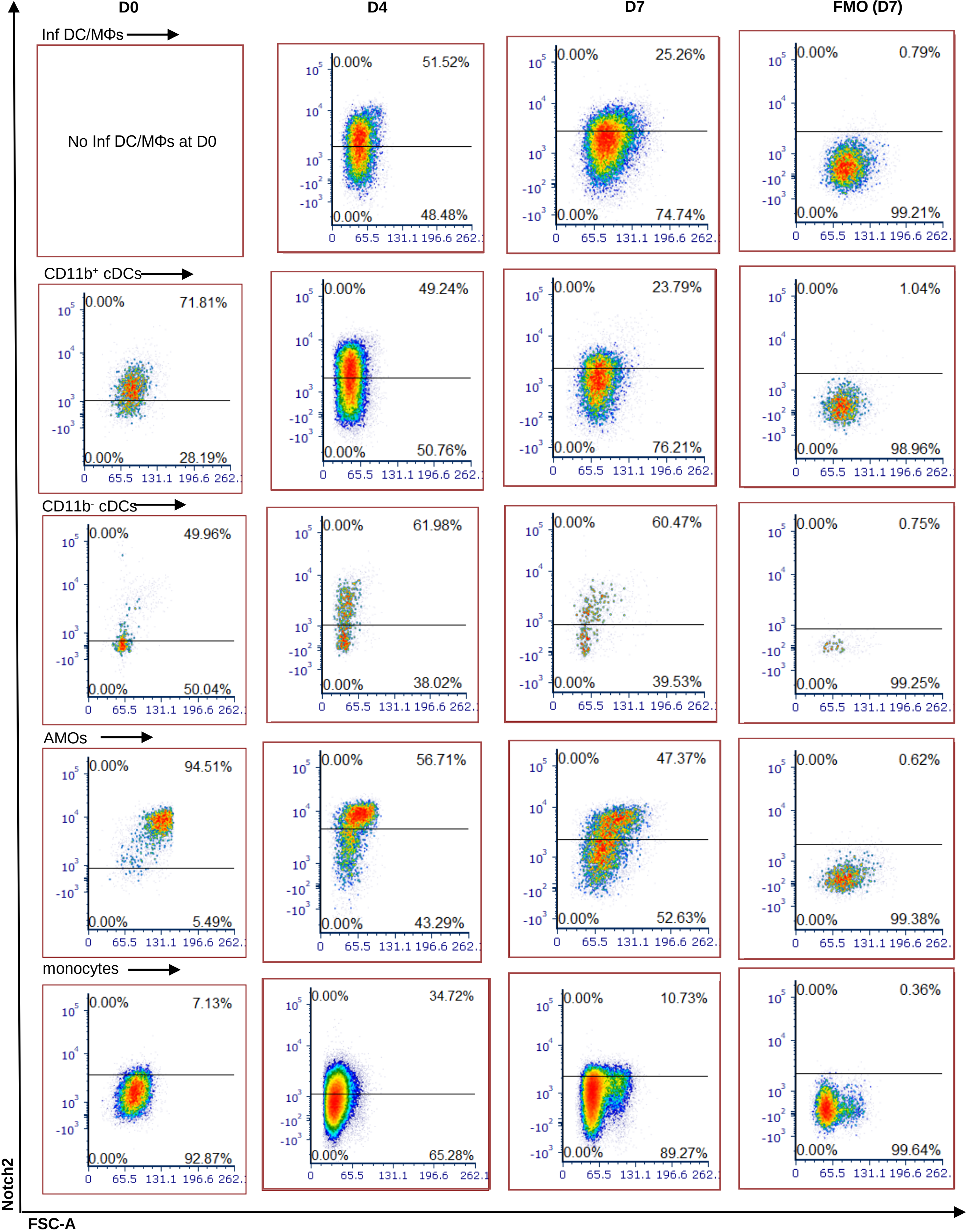

Figure S4. Representative plots of the percentage of Notch2 expressing cells among Inf DC/MΦs, CD11b⁺ cDCs, CD11b⁻ cDCs, AMOs, or monocytes in naïve lungs and days 4 as well as 7 PI lungs (n=3-6).
