# Supplementary material for: Notch regulates Histoplasma capsulatum clearance in mouse lungs during innate and adaptive immune response phases in primary infection
Source: J Leukoc Biol. 2022 May 23;112(5):1137–54. doi: 10.1002/JLB.4A1221-743R (PMC9613517; doi:10.1002/JLB.4A1221-743R)
Supplement: Supplementary file 5 — Figure S5. Representative plots of the percentage of NR expressing cells among IFNγ+ CD4+ T cells and IFNγ‐ CD4+ T cells in lungs and LNs at days 7 and 14 PI (n=4‐10). [file JLB-112-1137-s005.pdf]

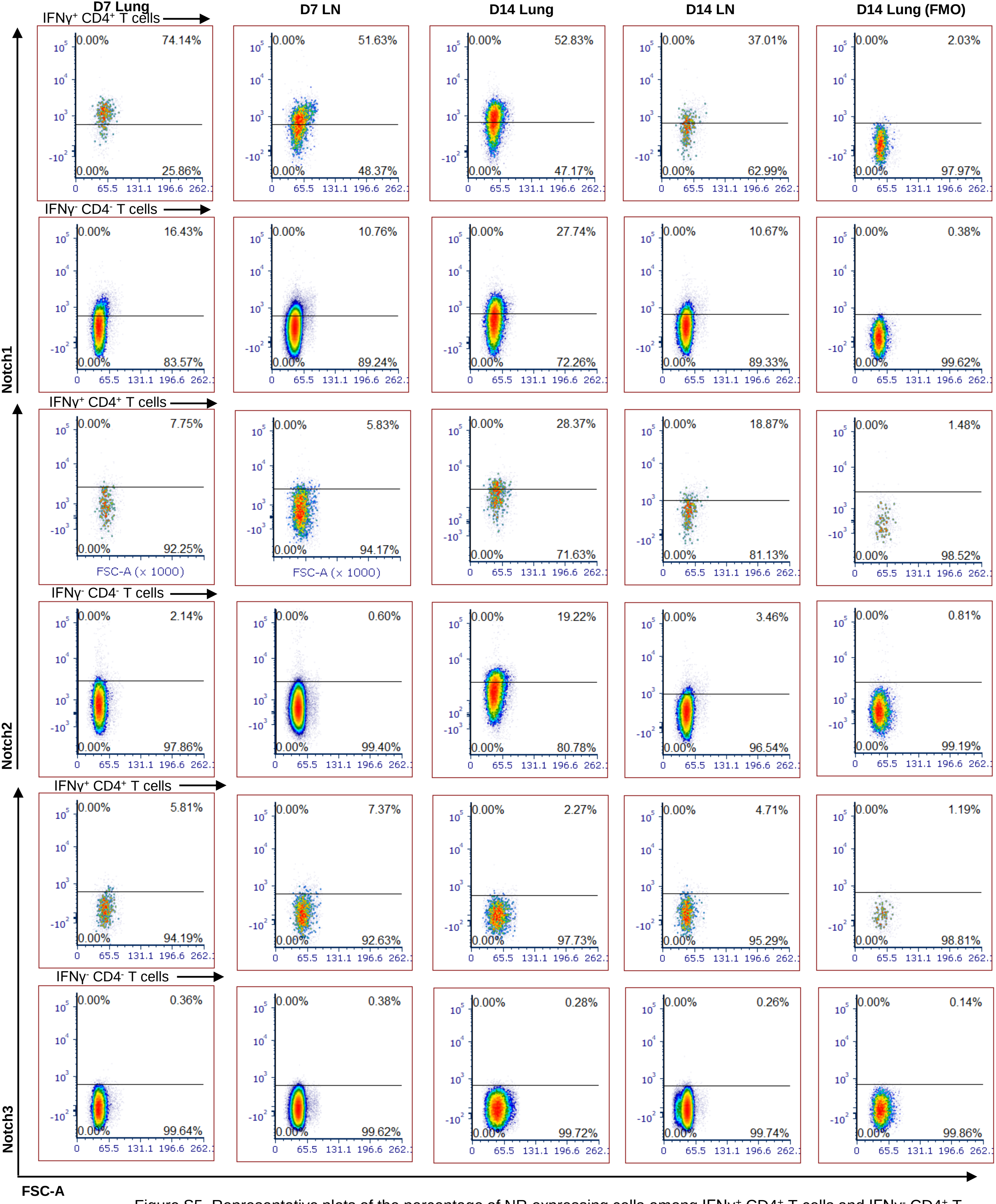

Figure S5. Representative plots of the percentage of NR expressing cells among IFNγ+ CD4+ T cells and IFNγ- CD4+ T cells in lungs and LNs at days 7 and 14 PI (n=4-10).
